# 268. Human Mesenchymal Stem Cells Augment Neutrophil Function to the Human Fungal Pathogen, *Candida albicans*

**DOI:** 10.1093/ofid/ofad500.340

**Published:** 2023-11-27

**Authors:** Daniel Floyd, Yuan Tan, Luciana Souza-Moreira, Shirley H J Mei, Kyle Timmer, Michael Mansour

**Affiliations:** Massachusetts General Hospital, Somerville, Massachusetts; Ottawa Hospital Research Institute, Ottawa, Ontario, Canada; Ottawa Hospital Research institute, Ottawa, Ontario, Canada; Ottawa Hospital Research Institute, Ottawa, Ontario, Canada; Massachusetts General Hospital, Somerville, Massachusetts; Massachusetts General Hospital, Somerville, Massachusetts

## Abstract

**Background:**

Neutrophils are critical innate immune cells that play a crucial role in protection against invading pathogens. Mesenchymal stem cells (MSC) are multipotent progenitors that can differentiate into osteoblasts, chondrocytes, and adipocytes and possess immunomodulatory effects. MSC are the focus of clinical investigation in various comorbidities, including diabetes, cancer, and invasive infections.

**Methods:**

Human MSC were derived from bone marrow and have been characterized to meet all criteria of MSC (ISCT guideline). Cells were seeded at 100,000 cells per well in fibronectin (50ug/mL)-coated 24-well plate and incubated for 24 hours at 37°C, 5% CO_2_. The next day, 0.5 mL of whole blood from three healthy donors was added for another 24 hours, after which the neutrophils were isolated, viability measured, and a high throughput multiparametric neutrophil function assay was performed, compared to non-MSC exposed neutrophils (control neutrophils). Functions tested included ROS (dihydrorhodamine 123 (DHR123), phagocytosis of iRFP-expressing *Candida albicans,* ectodomain shedding (loss of CD62L), and degranulation (increased expression of CD66b). Multiparametric assays were measured on a BD FACS Celesta 4 laser flow cytometer and data was analyzed on FlowJo software.

**Results:**

Neutrophils co-cultured with MSC had an average viability of 96.3% compared to control neutrophils at 92.15%. Using the multiparametric neutrophil functional assay, neutrophils exposed to MSC *ex vivo* had increased functional responses to the fungal pathogen *Candida albicans.* MSC-conditioned neutrophils exhibited a 33.2% increase in degranulation, a 13.4% increase in ROS generation, and 16.7% additional phagocytosis compared to non-MSC neutrophil (control). Compared to fresh neutrophils isolated the day of testing, MSC-conditioned neutrophils had an average increase in phagocytosis, degranulation, shedding, and ROS generation of 36.9%, 62.7%, 50.3%, and 37.8%, respectively.

**Conclusion:**

MSC increase the functional effector activity of neutrophils in healthy controls, specifically ROS generation, phagocytosis, ectodomain shedding, and degranulation in response to *C. albicans*. These data raise the possibility of MSC as a novel therapeutic intervention for patients with invasive infections.

**Disclosures:**

**Michael Mansour, MD, PhD**, Thermofisher: Grant/Research Support

